# Tracing the paths of modular evolution by quantifying rearrangement events of protein domains

**DOI:** 10.1186/s12862-024-02347-7

**Published:** 2025-01-08

**Authors:** Abdulbaki Coban, Erich Bornberg-Bauer, Carsten Kemena

**Affiliations:** 1 Institute for Evolution and Biodiversity, University of Münster, Münster, 48159 Germany; 2 Departement of Protein Evolution, Max Planck Institute for Biology Tübingen, Tübingen, 72076 Germany

**Keywords:** Protein domains, Evolution, Domain arrangements

## Abstract

**Background:**

Protein evolution is central to molecular adaptation and largely characterized by modular rearrangements of domains, the evolutionary and structural building blocks of proteins. Genetic events underlying protein rearrangements are relatively rare compared to changes of amino-acids. Therefore, these events can be used to characterize and reconstruct major events of molecular adaptation by comparing large data sets of proteomes.

**Results:**

Here we determine, at unprecedented completeness, the rates of fusion, fission, emergence and loss of domains in five eukaryotic clades (monocots, eudicots, fungi, insects, vertebrates). By characterizing rearrangements that were previously considered “ambiguous” or “complex” we raise the fraction of resolved rearrangement events from previously ca. 60% to around 92%. We exemplify our method by analyzing the evolutionary histories of protein rearrangements in (i) the extracellular matrix, (ii) innate immunity across Eukaryota, Metazoa, and Vertebrata, and (iii) Toll-Like-Receptors in the innate immune system of Eukaryota. In all three cases we can find hot-spots of rearrangement events in their phylogeny which (i) can be related with major events of adaptation and (ii) which follow the emergence of new domains which become integrated into existing arrangements.

**Conclusion:**

Our results demonstrate that, akin to the change at the level of amino acids, domain rearrangements follow a clock-like dynamic which can be well quantified and supports the concept of evolutionary tinkering. While many novel domain emergence events are ancient, emerged domains are quickly incorporated into a great number of proteins. In parallel, the observed rates of emergence of new domains are becoming smaller over time.

**Supplementary Information:**

The online version contains supplementary material available at 10.1186/s12862-024-02347-7.

## Background

Modularity plays an essential role in the evolution of proteins [1–3]. Domains are reusable units of protein evolution which, through their variable arrangements, provide a vast repertoire of structural and functional diversity. Therefore, tracking domain rearrangement events during evolution can provide valuable insights on the evolution of a particular protein or more complex cellular components.

So far, significant roles of domain rearrangements have been reported for gene and genome evolution [4, 5], the evolution of many complex molecular systems such as the blood coagulation system [6, 7] and protein networks like the extracellular matrix (ECM) [8].

Over the last two decades, the availability of large sets of genomes and proteomes facilitated the elucidation of domain rearrangement events across large phylogenies [9–12]. However, all of the studies provide only single-step solutions such as the fusion of two existing arrangements or the addition of a newly emerged domain to an existing arrangement. Furthermore, quantitative analyses were hampered by the availability of only a limited set of domain rearrangement events.

A single-step event to determine the evolutionary path of one domain arrangement into another is not sufficient in all cases. Especially when a long time has elapsed between two nodes of a phylogeny, several mutations may have already accumulated in a single domain arrangement. This is akin to analyzing protein sequences at the level of amino acids where a single difference at a given position may be the results of a single substitution or may have resulted from several successive substitution at the same site. Thus, a single-step event cannot resolve the evolutionary history anymore and therefore the complete evolutionary path of the domain arrangement cannot be determined.

**Fig. 1:**
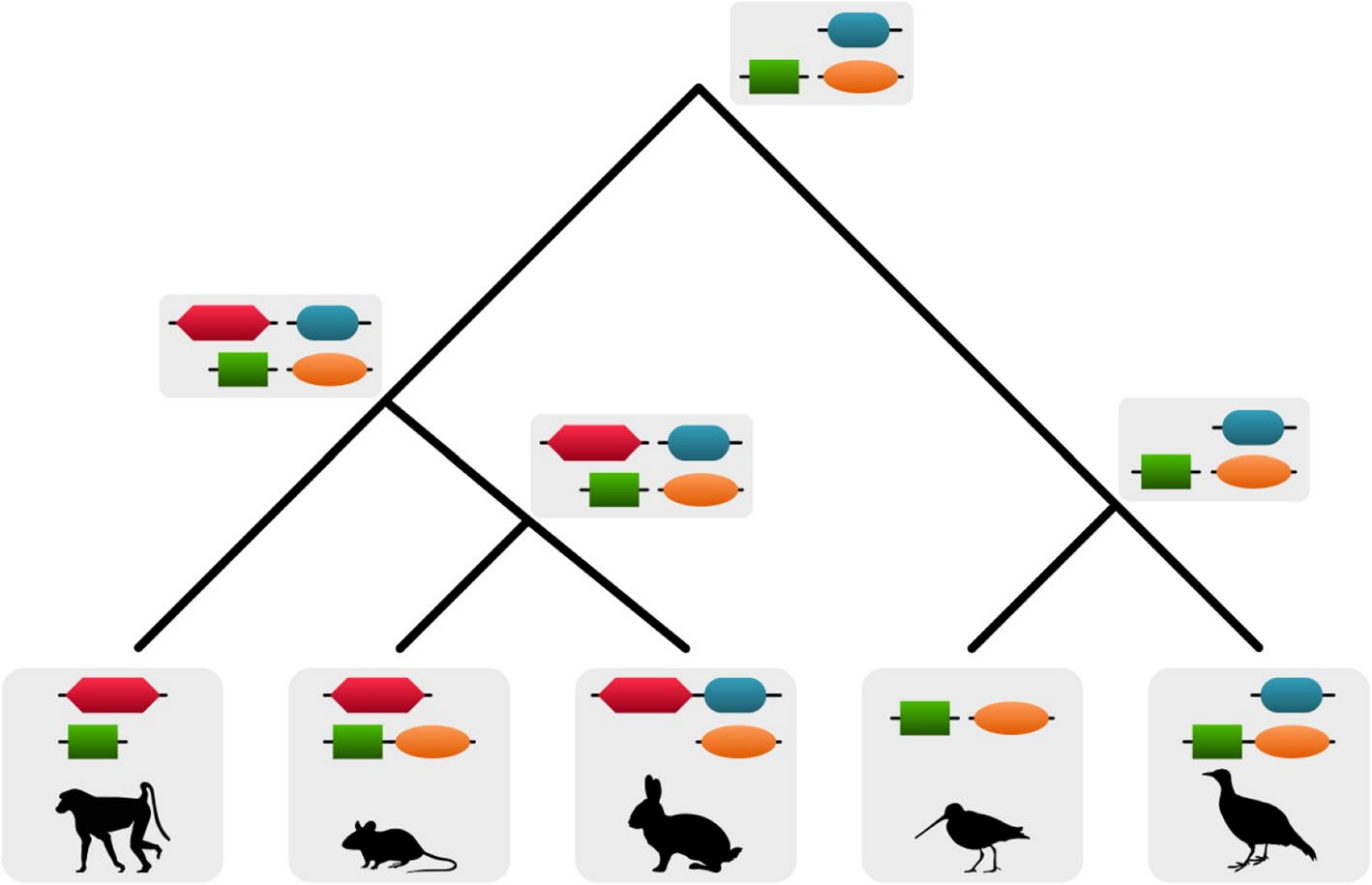
Phylogenetic tree showing examples of domain and domain arrangement emergences. A novel domain consists of many amino acids. The probability that this happens several times independently is rather low, therefore a domain is assumed to emerge only once. Domain arrangements on the other hand are much more volatile and can emerge several times independently (green/orange arrangement)

Consequently, to identify domain rearrangement events over the course of evolutionary history, it becomes necessary to carefully reconstruct ancestral domain arrangements and trace the changes on these arrangements along the descending lineages in a phylogeny (Fig. 1).

Moore et al. [11] and Dohmen et al. [12] successfully reconstructed ancestral domain arrangements and calculated the rates of domain rearrangement events across several eukaryotic clades. While the earlier study included the event types of fusion, fission, terminal domain loss and terminal domain gain, the latter study included further event types such as single domain emergence and single domain loss. Dohmen et al. [12] accomplished a rate of 50 to 70% identified rearrangement events on phylogenies of monocots, eudicots, fungi, insects and vertebrates, representing a time span of more than 1.5 billion years. They developed DomRates, which utilizes Fitch parsimony to uncover domain rearrangement events and Dollo parsimony to identify single domain emergences. Neither Moore et al. [11] nor Dohmen et al. [12] managed to characterize and quantify the domain rearrangement events where more than one possible solution exists (e.g. the arrangement AB may result from several alternative processes: A + B $\rightarrow$ AB and ABC - C $\rightarrow$ AB) and the multi-stepped events (e.g. B + C $\rightarrow$ BC, A + BC $\rightarrow$ ABC, ABC - C $\rightarrow$ AB).

Both earlier studies remained agnostic by marking these events as *ambiguous* and *complex* solutions, respectively. Besides, DomRates was not designed to track domain rearrangements of specific proteins, but calculates the rearrangement events in each node of the phylogeny.

In this study we present DomRates-Seq, which addresses the shortcomings mentioned above (large proportion of complex events). DomRates-Seq is designed to minimize ambiguity, increase resolution and track protein evolution in a given phylogeny. This is done by employing sequence similarity to resolve ambiguous cases. Furthermore, we introduce multi-step events to be able to solve many of the complex events that could not be solved by single-step events. Finally, DomRates-Seq also allows to track the evolutionary history of the domain arrangements step by step.

To demonstrate the improvement in terms of completeness compared to DomRates, we analyzed domain rearrangement events within five different eukaryotic clades (monocots, eudicots, fungi, insects, and vertebrates). To further show the effectiveness of DomRates-Seq, we provide three case studies: We track the evolution of human extracellular matrix and innate immunity proteins using domain rearrangement events. Lastly, we uncover the evolutionary history of 10 human TLR proteins.

## Methods

### Reconstruction of ancestral domain arrangements

As described in [12], DomRates relies on parsimony to determine the existence of domains and domain arrangements. In a first step we use Dollo parsimony to determine the available domain content at each node. Dollo parsimony allows a domain to emerge only once. That is reasonable because evolutionary domain convergence, i.e. the formation of the same domain from different sequences is extremely unlikely. Furthermore, Pfam domain sequences are clustered together assuming a single ancestor for a protein family. In a second step, Fitch parsimony is used to model the changes of domain arrangements in the ancestral nodes of the phylogeny. Different from Dollo parsimony, Fitch parsimony allows domain arrangements to appear independently. It has been shown that domain arrangements are more variable and can be recombined to form new domain arrangements, therefore possibly allowing as well the convergent evolution of the same domain arrangement. Both assumptions have been used before in different studies [11, 13]. For more details please see Supplementary Material 1. Studies, analyzing the amount of independently evolved domain arrangements, come to very different results ranging from 0.4% [14] to 25% [15]. The latter study is more recent and based on a much larger data set. Therefore, it is conceivable that a domain arrangement can evolve several times independently (Fig. 1).

DomRates uses a conservative approach on the emergence of single domains to keep single domain losses at minimum. When the same domain is only found in distantly related clades but nowhere in the middle, DomRates marks them as complex solutions. In this work however, DomRates-Seq marks the emergence at the last common ancestor and counts the missing domains in other clades as losses. By using complete implementation of Dollo parsimony, DomRates-Seq identifies possible origins of single domains instead of marking them unidentified. To highlight the differences between DomRates and DomRates-Seq methods, we test both on the origins of two single domains from monocots, PF01261 and PF13304. While DomRates infers multiple complex results, using Dollo parsimony principles, DomRates-Seq identifies last common ancestor (LCA) of the single domain in question (Supplementary Material 2).

**Fig. 2:**
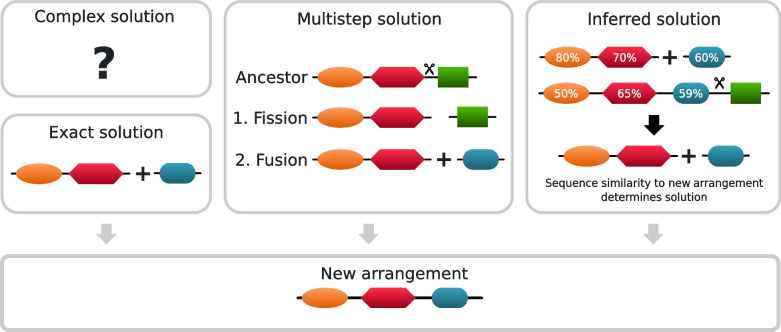
Schematic representation of defined solution types in DomRates-Seq. Exact solutions are solutions with a single-step event that can explain the new arrangement. DomRates-Seq now supports multi-step and inferred solutions. Multi-step solutions are solutions that combine multiple single-step events. Inferred solutions are ambiguous solutions as defined in DomRates (multiple possible single-step events) that can now be solved using sequence similarity. Every rearrangement event that cannot be explained by the algorithm is called complex solution

We also replace three solution types presented in DomRates with more accurate solutions. DomRates uses *exact, non-ambiguous, ambiguous,* and *complex* solutions. Here we introduce *inferred* and *multi-step* solutions to solve all the ambiguity resulting from *ambiguous* and *non-ambiguous* solutions and identify the very large proportion of the *complex* solutions. If there is an ambiguity with more than one possible domain rearrangement event, DomRates-Seq performs pairwise alignments between the domains of parent and child domain arrangements. Here, we perform pairwise alignments using the BLOSUM62 scoring matrix, however DomRates-Seq can be used with various scoring matrices. Considering a domain arrangement might emerge by multiple steps of rearrangement events, DomRates-Seq checks the possible multi-step events on every node in the phylogeny to provide more complete results (Fig. 2).

It is also possible to utilize DomRates-Seq for tracking down the evolutionary history of specific proteins. Instead of calculating the rates of domain rearrangement events, one can follow domain rearrangement events of a protein or a protein set step by step on a provided phylogenetic tree. The output files are designed to be used with iTOL [16] to generate a tree depicting domain rearrangement events.

### Dataset preparation and analyses

We prepared two proteome datasets. The comparison dataset was used to compare DomRates-Seq with the previous DomRates version. The second dataset (use case set) is used for example cases showing what kind of analyses can be done with DomRates-Seq. The example cases are proteins of the extra cellular matrix (ECM), innate immunity and TLR proteins.

One hundred seventy-four proteomes for the comparison dataset were downloaded from NCBI using the NCBI database tools [17]. Further 131 proteomes were downloaded for the ECM dataset. We cleaned proteomes from isoforms and retained the longest isoforms using a custom python script (provided with the study data). We used PfamScan v1.6 [18] with Pfam database v36 [19] to annotate domain arrangements. We assessed proteome quality using DOGMA v3.7 [20] and removed proteomes with the quality score smaller than 75%. For the comparison data set we ended up with 184 proteomes ( Supplementary Material 4) and for the ECM dataset with 107 proteomes (Supplementary Material 5). The proteomes of the comparison set were split into the following five clades: Monocots, Eudicots, Fungi, Insects, and Vertebrates as in [12]. Then we run DomRates v1.2 and DomRates-Seq with default parameters on each group.

The use cases data were analyzed as following: We run DomRates-Seq with -c option to track the evolutionary history of previously reported 323 ECM proteins (Supplementary Material 6) [8]. Further DomRates-Seq runs were performed for 306 innate immunity proteins ( Supplementary Material 7) downloaded from Uniprot [21] using the keyword *Innate immunity [KW-0399]* and taxonomy human and for all 10 TLR proteins (Supplementary Material 8).

## Results

### Improvements on DomRates

With the introduction of the sequence based methods (i.e pairwise alignments), the ambiguity of the DomRates results are resolved.

**Fig. 3:**
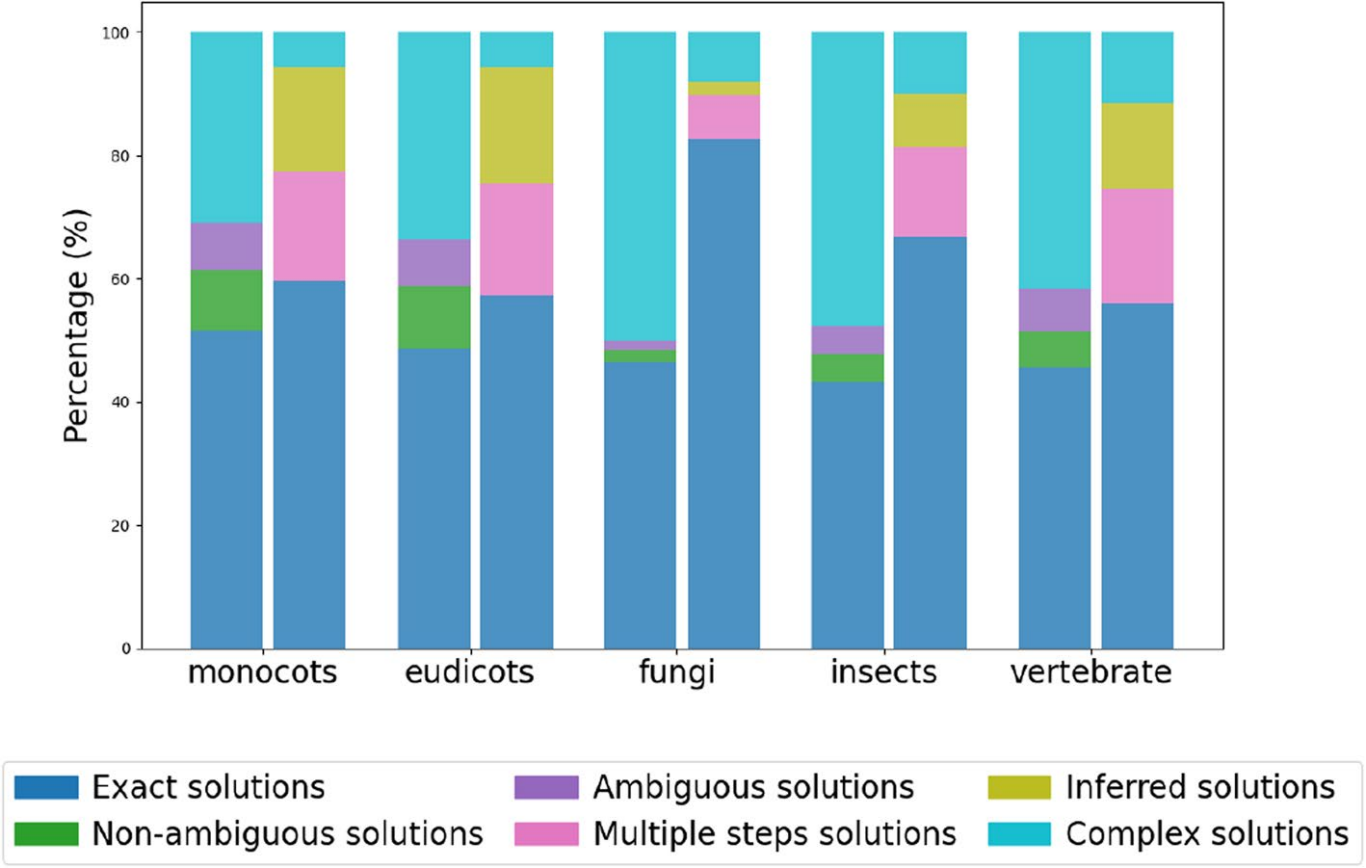
Proportion of solution types of DomRates (left bars) and DomRates-Seq (right bars)

In addition to resolving all ambiguous and non-ambiguous solutions defined in DomRates, we are also able to between 73,5% (eudicots) and 84,4% (arthropods) of all complex solutions in the five studied clades. The fraction of unexplained solutions reduces to 5,75% - 11,53% compared to 30,88% - 50,14% in DomRates (Fig. 3).

**Fig. 4:**
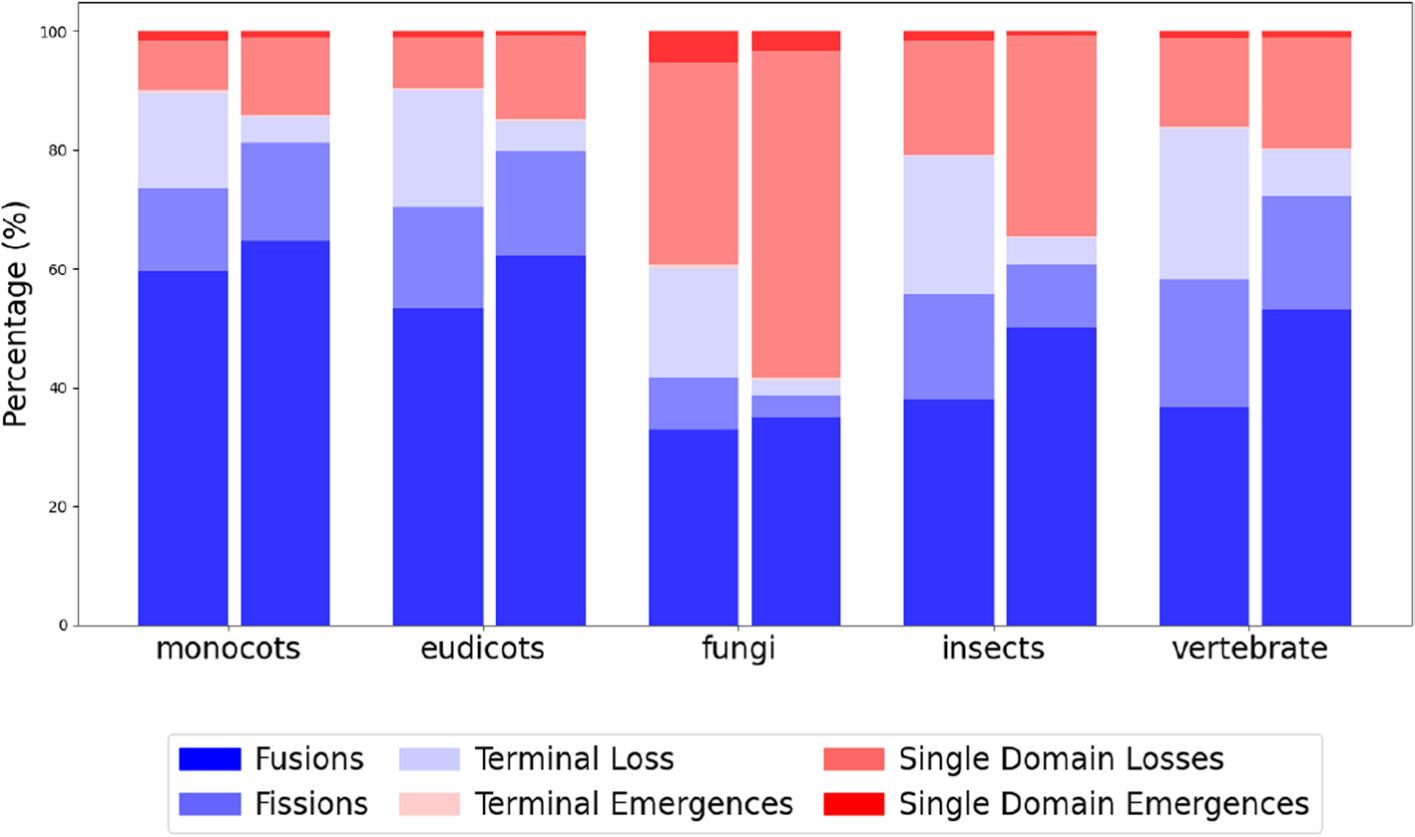
Comparison of the event proportions in DomRates (left bars) and DomRates-Seq (right bars) in five different clades

Besides the increased resolution in terms of defined solutions, the total number of explained domain rearrangement events are also increased almost 2.5-fold (57564 to 135455) in DomRates-Seq, compared to DomRates. While the percentage of fusion and fission events stay relatively similar (44.1% vs 53.0% and 15.81% vs 13.52%, respectively), the percentage of the single domain losses and terminal loss events change drastically (from 17.0% to 26.9% and 20.5% to 4.91%). This change is due to the complete implementation of Dollo parsimony (Fig. 4, Table 1). DomRates is designed to be conservative in terms of the origin of the single domains. When a single domain is found in remote branches in a phylogeny, it marks the single domain occurrences as *complex solution* to keep the number of domain losses at a minimum within the phylogeny. With the complete implementation of the Dollo parsimony in DomRates-Seq, we accurately identify the origins of the single domains in the phylogeny (Supplementary Material 2).

**Table 1:** Comparison of DomRates and DomRates-Seq results

Clade	Version	Fusion	Fission	Terminal loss	Terminal gain	Single loss	Single gain
Monocots	DomRates	2110 (59.72%)	485 (13.73%)	568 (16.08%)	17 (0.48%)	295 (8.35%)	58 (1.64%)
	DomRates-Seq	4243 (64.76%)	1078 (16.45%)	286 (4.37%)	20 (0.31%)	856 (13.06%)	69 (1.05%)
Eudicots	DomRates	2786 (53.26%)	895 (17.11%)	1028 (19.65%)	26 (0.50%)	444 (8.49%)	52 (0.99%)
	DomRates-Seq	6182 (62.20%)	1738 (17.49%)	517 (5.20%)	27 (0.27%)	1402 (14.11%)	73 (0.73%)
Fungi	DomRates	3104 (32.86%)	838 (8.87%)	1727 (18.28%)	77 (0.82%)	3208 (33.96%)	493 (5.22%)
	DomRates-Seq	8990 (34.86%)	992 (3.85%)	668 (2.59%)	113 (0.44%)	14129 (54.78%)	898 (3.48%)
Insects	DomRates	6650 (37.94%)	3127 (17.84%)	4063 (23.18%)	49 (0.28%)	3374 (19.25%)	263 (1.50%)
	DomRates-Seq	22704 (50.10%)	4844 (10.69%)	2065 (4.56%)	70 (0.15%)	15290 (33.74%)	346 (0.76%)
Vertebrates	DomRates	8013 (36.71%)	4691 (21.49%)	5524 (25.31%)	80 (0.37%)	3258 (14.93%)	261 (1.20%)
	DomRates-Seq	25398 (53.07%)	9163 (19.15%)	3737 (7.81%)	95 (0.20%)	9003 (18.81%)	459 (0.96%)

### Extracellular matrix evolution

The extracellular matrix (ECM) is a hallmark of metazoans, composed of a network of self-assembling fibrous proteins and closely associated molecules. While the ECM provides mechanical support for neighboring tissues with its fibrous proteins, it also serves as a medium in which cells communicate, differentiate and regenerate [22]. Since its composition is highly differentiated between various tissues, it is a perfect model to investigate how domain evolution patterns can differ within a group of functionally similar proteins.

Of 323 studied ECM proteins, PfamScan annotated domain arrangements of 313 proteins. 180 novel domain arrangements are found within the 313 proteins. While most (132) of the domain arrangements are found only once within ECM proteins, there are multi-copy domain arrangements: PF00110 (18) and PF01471 PF00413 PF00045 (13) (Supplementary Material 9). We identify 177 distinct domains within ECM proteins of which 14 are unique to proteins involved in the ECM.

Most of the human ECM proteins are well conserved within vertebrates. We identify at least three hotspots on the evolutionary history of ECM proteins where the number of events is substantially elevated compared to the other nodes in the phylogeny: The roots of Eukaryota, Metazoa and Vertebrata having 40, 26 and 44 distinct solutions, respectively. Similar to the high number of solutions identified in these nodes, the highest numbers of single domain emergences are found in the LCAs of Eukaryota and Metazoa: 40 and 19, respectively. The roots of Bilateria, Holozoa, and Vertebrata have also high numbers of single domain emergences: 12, 10, and 8, respectively. The high numbers of novel domain emergences at these nodes underscore the significant impact of evolutionary innovation on the emergence of novel clades. In total, we identify 127 fusion, 23 fission, 9 terminal domain loss, 9 terminal domain emergence, and 110 single domain emergence events across the phylogeny. Overall, we identify 220 solutions and 278 events (Supplementary Material 3).

The identified 110 newly emerged single domains eventually incorporate into 90.1% (282/313) of the ECM proteins. The roots of Eukaryotes and Metazoa are especially important, providing 53.6% of the single domain emergences, 40, and 19 emergence events, respectively.

### Human innate immunity evolution

Innate immunity is the first line of defense against pathogens, consisting of proteins recognizing and responding to pathogens. Similar to the ECM, its high diversity makes it an adequate system to study domain evolution through various species spanning around 1 billion years [23].

We follow the same approach for the human innate immunity proteins as in the analysis of ECM evolution. Of 306 innate immunity proteins studied, PfamScan is able to annotate domain arrangements of 293 proteins. We identify 230 unique domain arrangements and 299 domains with 18 unique to innate immunity proteins (Supplementary Material 10).

Analyzing the rearrangements during the evolution of innate immunity across Eukaryota, we identify 270 unique solutions and 307 events. Similar to the ECM evolution, the roots of Eukaryota, Metazoa and Vertebrata are found to be evolutionary hotspots comprising more than half of the identified solutions (58,1%), 94, 23 and 40, respectively. In total, we identify 148 unique single domain emergences which are incorporated into 232 (75,82%) innate immunity proteins. Similar to overall solution counts, the root of Eukaryota, Metazoa and Vertebrata have the highest number of single domain emergences (91, 13 and 11, respectively).

### Evolution of human TLR proteins

**Fig. 5:**
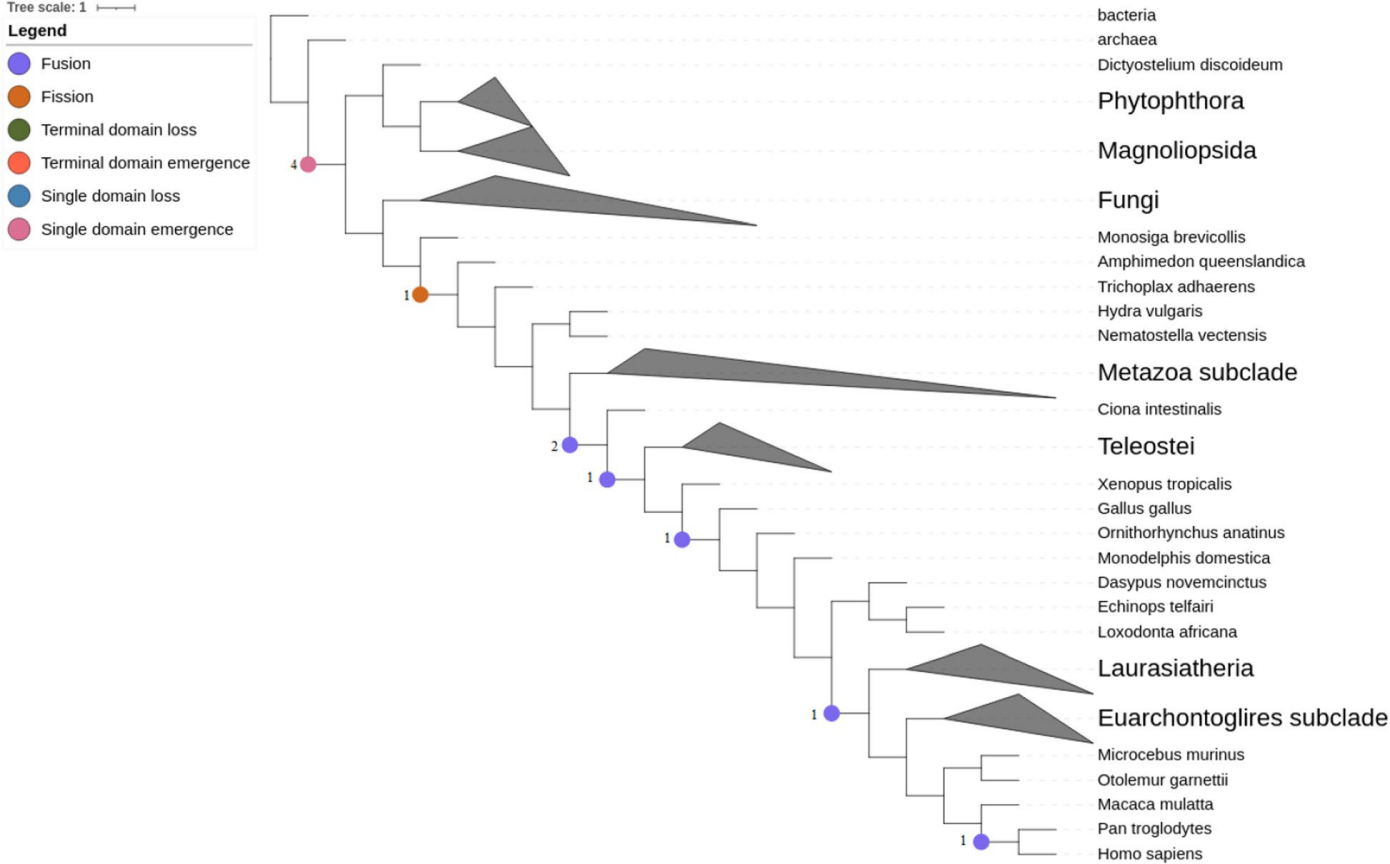
Domain rearrangement events during TLR evolution

Human TLR proteins consist of seven unique domain arrangements with combinations of eight domains. During the evolution of human TLR proteins we identify 6 fusion, 1 fission and 4 single domain emergence events. All of the identified domain emergences are found at the LCA of eukaryotes: TIR (PF01582), LRRNT (PF01462), LRR_6 (PF13516), and LRR_4 (PF12799). While half of the domains emerged at the LCA of Eukaryotes, the rest of the domains have emerged earlier in the tree. We identify an increase of events within the vertebrate clade, indicating the differentiation of the respective proteins (Fig. 5).

## Discussion

In this study we infer domain rearrangement events in five eukaryotic clades and track the domain rearrangement events in the evolutionary history of ECM, innate immunity and TLR proteins using DomRates-Seq, an improved version of the previously published tool DomRates [12]. With the new algorithm, we not just increase the resolution of the overall completeness of the software, but also provide another feature: DomRates-Seq now allows to track domain rearrangement events of a protein set instead of just calculating the overall domain rearrangement rates.

Using DomRates-Seq, most of the ambiguous, non-ambiguous, and complex solutions presented in DomRates are resolved as unique solutions and the total number of explained events is greatly increased. While in DomRates 40%−60% of the solutions were identified as complex and ambiguous solutions, DomRates-Seq resolves all ambiguous solutions and only 5%−10% of the solutions remain as complex solutions (Fig. 3). The increased number of resolved solutions provides crucial insights on the evolution of the studied clades. Complex solutions are generally artifacts of low resolution in the studied group of species. If a domain arrangement underwent many changes, even the allowed multi-step events might not be sufficient to create a link between the parent the child node of the phylogeny. Therefore, since some solutions remain unidentified (i.e. complex solutions), expanding the phylogeny would be useful to decrease the number of complex solutions.

Akin to previous reports [3, 10, 24], fusion events are found to be the most prevalent in every clade, except in fungi where single domain losses are higher, as also observed previously in [4, 12, 25].

We also trace domain rearrangement events on the evolutionary history of human ECM and innate immunity proteins. As for ECM, we identify three evolutionary hotspots where the number of events increased compared to the other nodes on the phylogenic tree: The roots of Eukaryota, Metazoa and Vertebrata.

Our results agree with the findings of [8] reporting three evolutionary hotspots (Eukaryota, Metazoa and Vertebrata) for the evolution of ECM proteins. This congruence of findings demonstrates the usefulness of a domain based approach which is coarse grained compared to sequence based approaches because fewer building blocks are considered and, therefore, computational efforts are significantly lower. As discussed in [8], “old domains” are rearranged into novel protein arrangements to give rise to the vast majority of the ECM proteins. Therefore, DomRates-Seq can be considered a practical tool to analyze domain rearrangement events on a large phylogeny. DomRates-Seq not only dramatically accelerates the process of analyzing domain rearrangements, but also offers profound insights into the modular evolution of proteins, paving the way for deeper understanding of the evolution of proteins.

Similar patterns of rearrangement events culminating at deep nodes in a phylogeny are also observed in the evolution of innate immunity. The LCAs of Eukaryota, Metazoa, and Vertebrata have the highest number of domain rearrangement events and the highest number of single domain emergences. Even though both ECM and innate immunity evolutions are heavily dependent on the domains evolved at the LCA of eukaryotes, a greater proportion of the innate immunity domains have originated at the LCA of eukaryotes. This emphasizes the high rates of invention needed for the immunity in eukaryotes.

Domains of relatively young age (i.e domains which originated in vertebrates) did also spread across different proteins during innate immunity evolution: 11 newly emerged domains at the LCA of vertebrates are shared by 25 proteins of 306 proteins in the innate immune system.

As for the evolution of human TLR proteins, we identify the origins of half (4/8) of the domains belonging to the TLR family proteins at the root of Eukaryota. The origins of the rest of these domains can be traced to deeper nodes in the phylogeny. Since TLR family proteins are diverse and found in many basal organisms [26–29], it is not surprising that we found the origin of the domains deep in the tree.

Akin to the evolution of the ECM and the innate immunity, step by step fusion events of TLR proteins with an ancient set of domains during the vertebrate evolution shaped the final structure of TLR family proteins in vertebrates.

At the roots of Eukaryota and Metazoa, high levels of single domain rearrangement events can be found. Given the increased rate of gene and genome expansions, duplications, and fusion events in these nodes [30–32], the significant number of domain rearrangement events identified on the LCAs of Metazoa and Vertebrata further underscores their accelerated evolutionary rates.

Even though the highest number of solutions can be assigned to the root of Vertebrata, the number of single domain emergences is relatively low. Most of the single domain emergences took place at the root of Eukaryota and Metazoa. During the later stages of ECM and innate immunity evolution, the domains rearranged into novel domain arrangements. This pattern reflects the nature of evolutionary tinkering: Evolution acts as an inventor early in the evolutionary timeline but when more sophisticated molecules are needed, evolution shifts towards refining and optimizing existing compounds rather than creating new ones.

## Conclusion

DomRates-Seq proves to be a valuable tool for a domain-based approach to study molecular evolution. DomRates-Seq is able to resolve ambiguities between alternative solutions as they were defined in the earlier version of DomRates with much greater resolution. Besides, compared to the previously reported studies, DomRates-Seq accurately infers domain rearrangement events in the studied phylogenies.

This study emphasizes that protein evolution relies to a large extent on the rearrangements of existing structures. The emergence of novel domains is more prevalent earlier in the evolutionary history, while proteins reusing existing structures become more prevalent during the later stages of evolution. Moreover, when new modules emerge during evolution, they may spread rapidly across different proteins and species. This rapid spread emphasizes the efficiency of evolutionary tinkering, where useful innovations are quickly adopted and integrated, demonstrating nature’s remarkable capacity to innovate and adapt through a mix of existing and novel components.

## Supplementary Information

Supplementary Material 1. Dollo and Fitch parisimony - This figure from Dohmen et al. [12] describes the Dollo and Fitch parsimony method to determine domain and domain arrangement content for each node.

Supplementary Material 2. New Dollo implementation - This figures shows an example how he new dollo implementation influences the results.

Supplementary Material 3. DomRates-Seq results for ECM - Results of the DomRates-Seq analysis of the ECM data set, showing the different events on the tree.

Supplementary Material 4. Comparison data set - Species set used for the comparison of DomRates with DomRates-Seq. Information contained are: Clade, Species, Accession ID and Dogma score.

Supplementary Material 5. ECM data set - Species set used for the extracellular matrix analyiss. Information contained are: Clade, Species, Accession ID and Dogma score.

Supplementary Material 6. : ECM Proteins - List of ECM proteins used for the analysis.

Supplementary Material 7. Innate Immunity Proteins - List of innate immunity proteins used for the analysis.

Supplementary Material 8. TLR Proteins - List of TLR proteins used for the analysis.

Supplementary Material 9. ECM domain arrangements - List of ECM proteins and their domain arrangements.

Supplementary Material 10. Innate immunity domain arrangements - List of innate immunity proteins and their domain arrangements.

## Data Availability

The developed software DomRates-Seq is available via the project website http://domainworld.uni-muenster.de/programs/domrates-Seq/ and with full source code on our gitlab https://zivgitlab.uni-muenster.de/domain-world/domratesseq. The datasets generated and/or analysed during the current study are available in the Zenodo repository, https://zenodo.org/records/13329608. DomRates-Seq is implemented in C++ and platform independent, published under the free GNU GPL licence version 3. For further information please check the UserManual on the website, where you can find also tutorials and example data sets to test the software.
