# Supplementary figures and images for: Tracing the paths of modular evolution by quantifying rearrangement events of protein domains
Source: BMC Ecol Evol. 2025 Jan 8;25:6. doi: 10.1186/s12862-024-02347-7 (PMC11707847; doi:10.1186/s12862-024-02347-7)

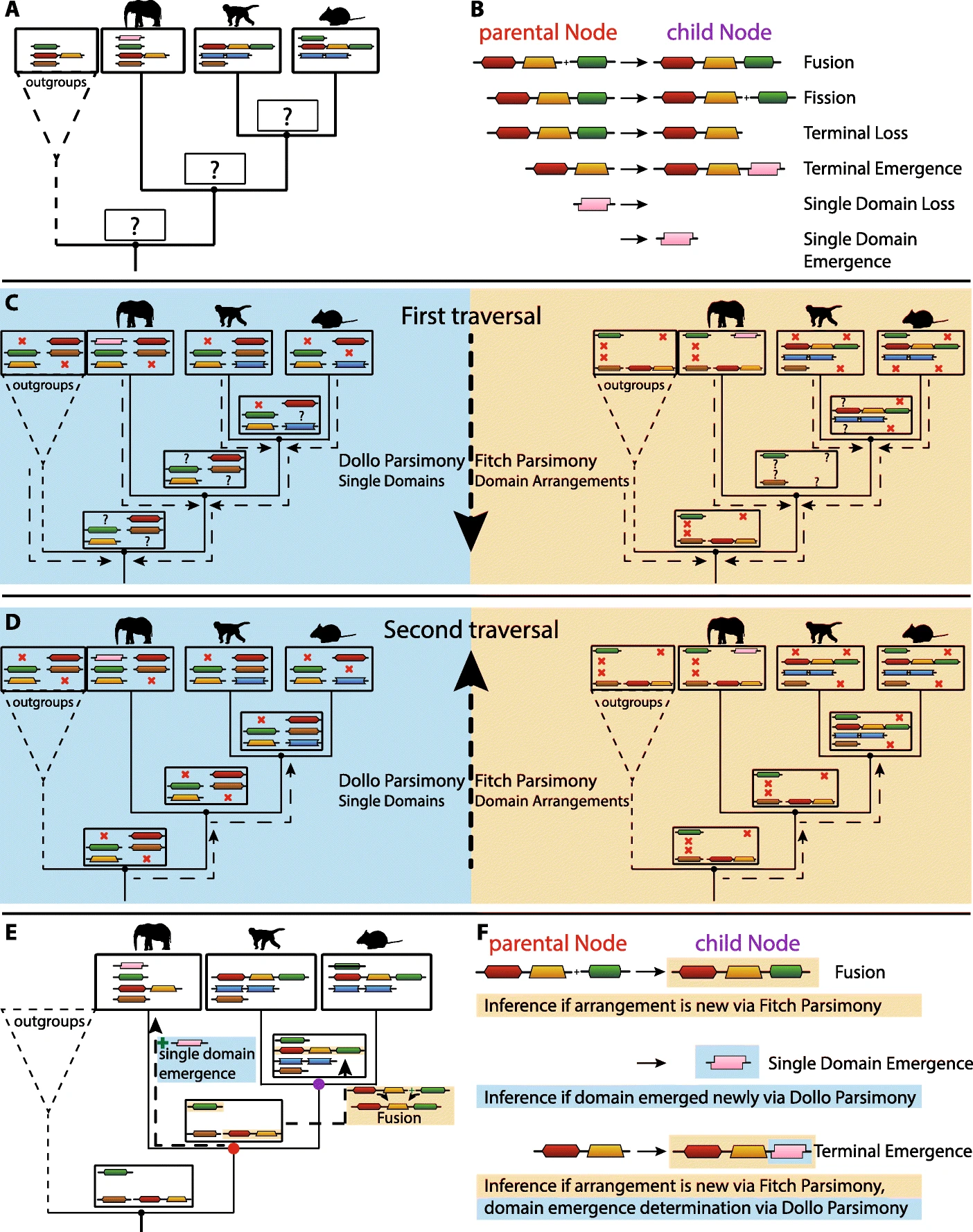

Supplement: Supplementary file 1 — Supplementary Material 1. Dollo and Fitch parisimony - This figure from Dohmen et al. [12] describes the Dollo and Fitch parsimony method to determine domain and domain arrangement content for each node. [file 12862_2024_2347_MOESM1_ESM.tiff]

a) PF01261    ● Complex    ● Maintained    ● Loss

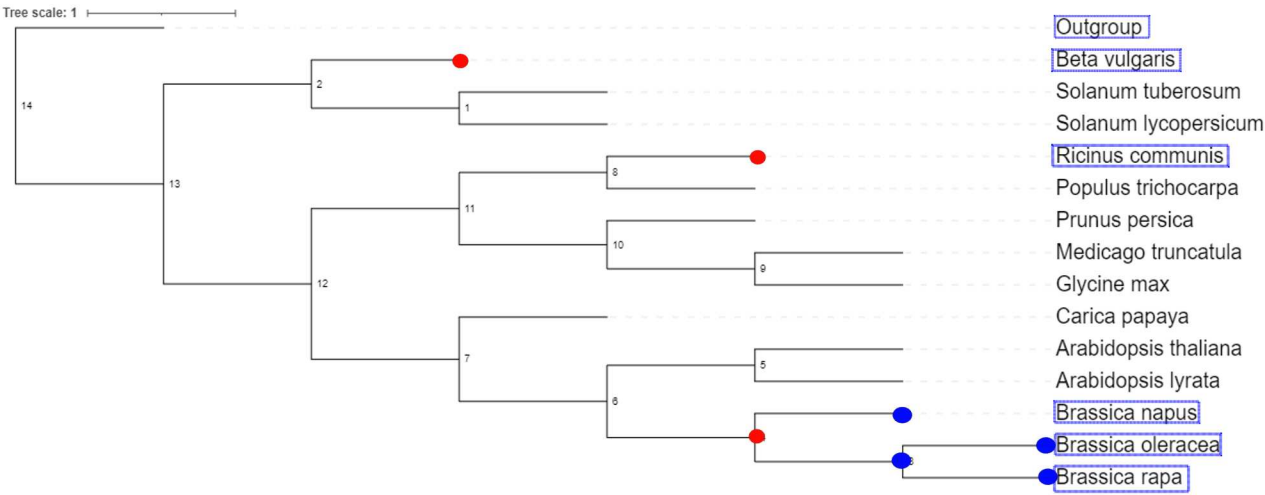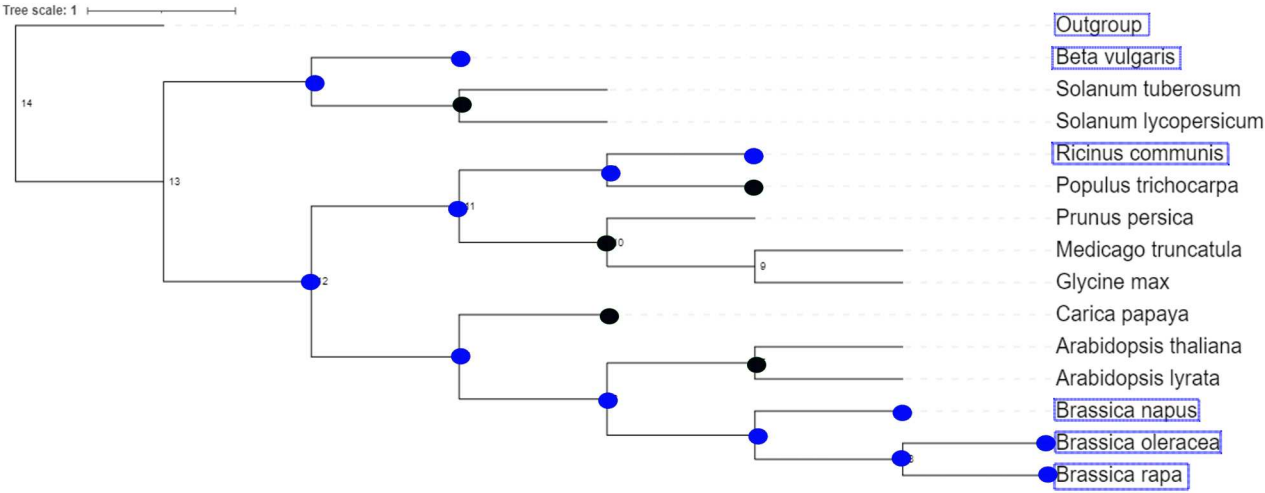

b) PF13304

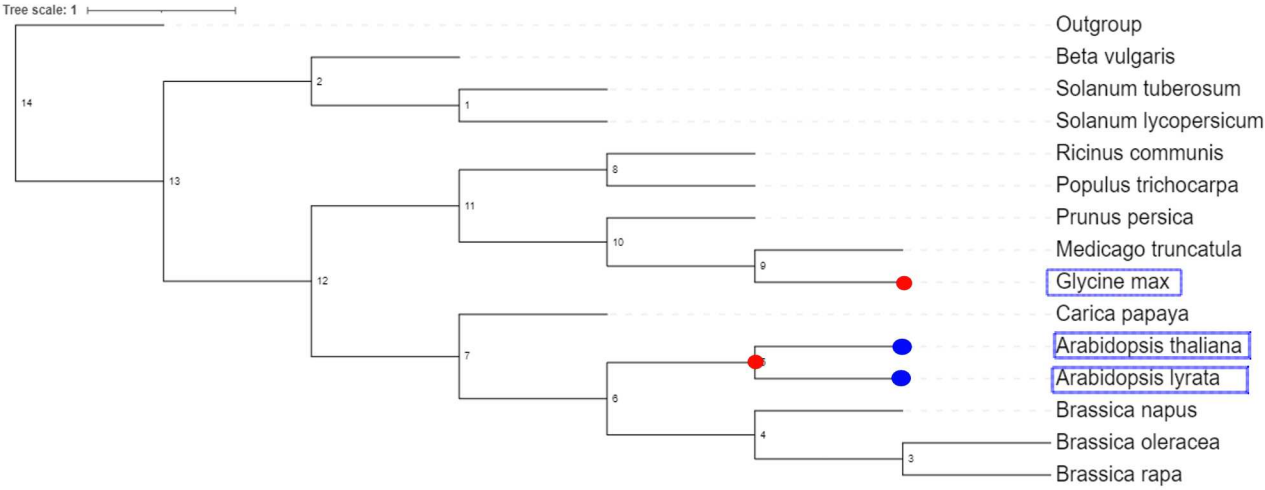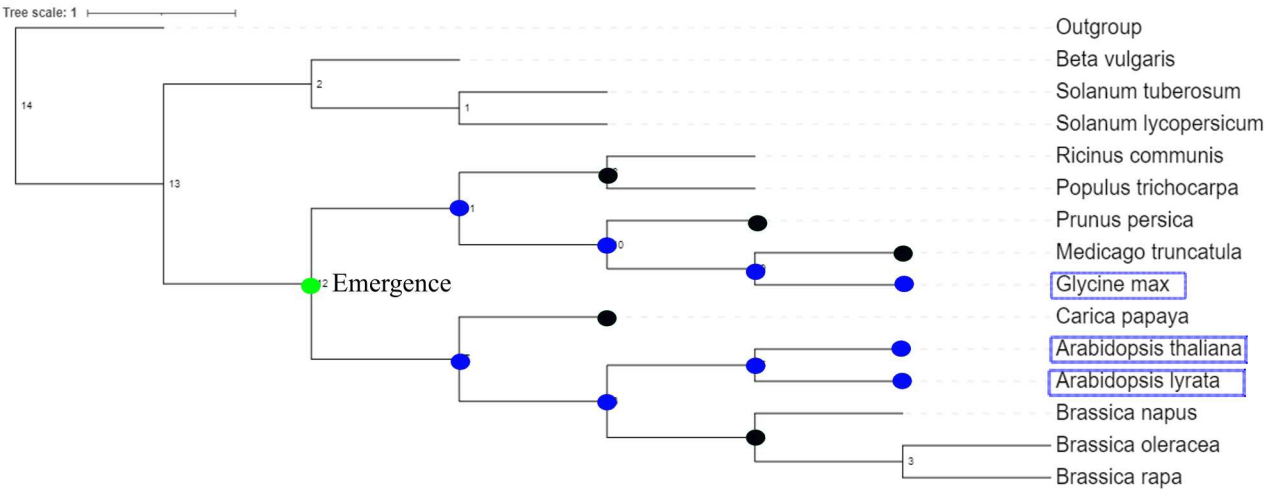

Supplement: Supplementary file 2 — Supplementary Material 2. New Dollo implementation - This figures shows an example how he new dollo implementation influences the results. [file 12862_2024_2347_MOESM2_ESM.pdf]
