# Supplementary material for: Tracing the paths of modular evolution by quantifying rearrangement events of protein domains
Source: BMC Ecol Evol. 2025 Jan 8;25:6. doi: 10.1186/s12862-024-02347-7 (PMC11707847; doi:10.1186/s12862-024-02347-7)
Supplement: Supplementary file 3 — Supplementary Material 3. DomRates-Seq results for ECM - Results of the DomRates-Seq analysis of the ECM data set, showing the different events on the tree. [file 12862_2024_2347_MOESM3_ESM.pdf]

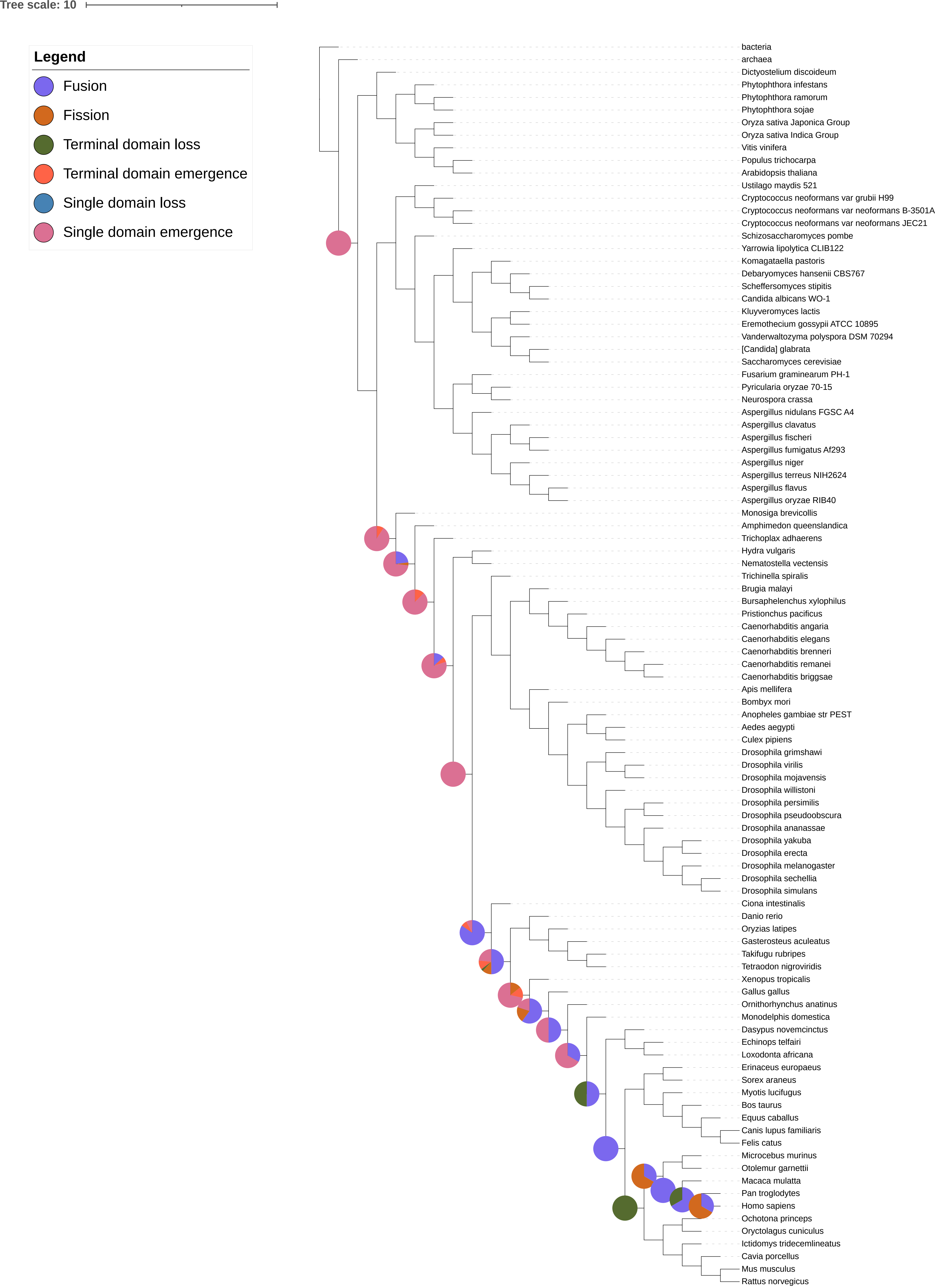
Tree scale: 10
Legend
Fusion
Fission
Terminal domain loss
Terminal domain emergence
Single domain loss
Single domain emergence
bacteria
archaea
Dictyostelium discoideum
Phytophthora infestans
Phytophthora ramorum
Phytophthora sojae
Oryza sativa Japonica Group
Oryza sativa Indica Group
Vitis vinifera
Populus trichocarpa
Arabidopsis thaliana
Ustilago maydis 521
Cryptococcus neoformans var grubii H99
Cryptococcus neoformans var neoformans B-3501A
Cryptococcus neoformans var neoformans JEC21
Schizosaccharomyces pombe
Yarrowia lipolytica CLIB122
Komagataella pastoris
Debaryomyces hansenii CBS767
Scheffersomyces stipitis
Candida albicans WO-1
Kluyveromyces lactis
Eremothecium gossypii ATCC 10895
Vanderwaltozyma polyspora DSM 70294
[Candida] glabrata
Saccharomyces cerevisiae
Fusarium graminearum PH-1
Pyricularia oryzae 70-15
Neurospora crassa
Aspergillus nidulans FGSC A4
Aspergillus clavatus
Aspergillus fischeri
Aspergillus fumigatus Af293
Aspergillus niger
Aspergillus terreus NIH2624
Aspergillus flavus
Aspergillus oryzae RIB40
Monosiga brevicollis
Amphimedon queenslandica
Trichoplax adhaerens
Hydra vulgaris
Nematostella vectensis
Trichinella spiralis
Brugia malayi
Bursaphelenchus xylophilus
Pristionchus pacificus
Caenorhabditis angaria
Caenorhabditis elegans
Caenorhabditis brenneri
Caenorhabditis remanei
Caenorhabditis briggsae
Apis mellifera
Bombyx mori
Anopheles gambiae str PEST
Aedes aegypti
Culex pipiens
Drosophila grimshawi
Drosophila virilis
Drosophila mojavensis
Drosophila willistoni
Drosophila persimilis
Drosophila pseudoobscura
Drosophila ananassae
Drosophila yakuba
Drosophila erecta
Drosophila melanogaster
Drosophila sechellia
Drosophila simulans
Ciona intestinalis
Danio rerio
Oryzias latipes
Gasterosteus aculeatus
Takifugu rubripes
Tetraodon nigroviridis
Xenopus tropicalis
Gallus gallus
Ornithorhynchus anatinus
Monodelphis domestica
Dasypus novemcinctus
Echinops telfairi
Loxodonta africana
Erinaceus europaeus
Sorex araneus
Myotis lucifugus
Bos taurus
Equus caballus
Canis lupus familiaris
Felis catus
Microcebus murinus
Otolemur garnettii
Macaca mulatta
Pan troglodytes
Homo sapiens
Ochotona princeps
Oryctolagus cuniculus
Ictidomys tridecemlineatus
Cavia porcellus
Mus musculus
Rattus norvegicus
